# Optimal Transport on Weighted Networks for Different Node Delivery Capability Schemes

**DOI:** 10.1155/2013/378083

**Published:** 2013-12-30

**Authors:** Fei Shao

**Affiliations:** ^1^Jiangsu Information Analysis Engineering Laboratory, Jinling Institute of Technology, Nanjing 211169, China; ^2^School of Information Technology, Jinling Institute of Technology, Nanjing 211169, China

## Abstract

Many real networks can be best described by weighted networks with a diversity of interactions between nodes measured by the weights of the edges. It is of great importance to improve the overall capacity of these real-world networks. In this paper, the traffic capacity of weighted network is investigated based on three different node delivery capability schemes: the delivery capacity of each node is constant in the first scheme while in the second and third schemes it is proportional to its node degree and node strength. It is shown by simulations that the network transfer capacity depends strongly on the tunable parameter. And different tunable parameter is suitable for different node delivery capability.

## 1. Introduction

The past few years have witnessed the emergence of interest in the network topological structure since the seminal study on small world networks by Watts and Strogatz [1] and on scale-free networks by Barabási and Albert [2]. Lots of biological, social, and communication systems can be viewed as complex networks while nodes represent individuals and edges represent the relationships between them. The previous studies on networks have been primarily focused on unweighted networks, edges between nodes are either present or not, represented as binary states. However, the gathering complete data of real networks display the variation of the strength of the edges between nodes, providing a more complete representation of real network structures. The analyses of the mobile communication networks [3], the scientific collaboration networks [4], the cellular metabolism [5], the world-wide airport network [6], and the Internet [7] have revealed that networks are not only specified by their topology but also by the dynamics of weight (such as the capacity and the intensity) taking place along the edges.

Lots of models have been presented to describe those real-world networks. The YJBT model [8] is a model for weighted scale-free network whose topology is the same as that of the BA model. Moreover, both the topology and the weights are driven by the connectivity according to the preferential attachment rule. The ZTZH model [9] is a generalization of the YJBT model incorporating a stochastic scheme for weight assignments based on both the degree and the fitness of node. And the AK model [10], in which the structural growth of the network is coupled with the edge weights, focus on a strength driven attachment instead of degree preferential attachment. In those models discussed above, the weight of edge is assigned when the edge is added and remains fixed thereafter. But actually, the addition of new nodes or edges will affect the weight of the existing edges. The BBV model [11] is proposed to describe the weights' reinforcement phenomenon which is triggered only by new added nodes. Wang model [12] is a traffic-driven evolution model where weights of edges between the existed nodes will also be rearranged.

Recently, the study of the network overall transfer capacity is becoming increasingly important due to the constantly growing significance of large communication networks such as the Internet. Finding optimal routing strategies to improve the transfer capacity is gaining increasing concern. Some are based on global information: the shortest path routing strategy [13], the efficient path routing strategy [14], and the generalized minimum information path routing strategy [15]; some are based on local information [16–20].

In this paper, we propose a novel routing strategy in which packets are transferred through the path based on the weight of edges with a tunable parameter *α*. To maximize the overall network transfer capacity which can be measured by the critical packet generating rate *R*_*c*_, the optimal tunable parameter *α* is achieved.

This paper is organized as follows. In Section 2 we describe the model and our routing strategy, followed by the experimental evaluations on BBV weighted networks and real world network in Section 3. The conclusions are given in Section 4.

## 2. Model

In BBV networks, the topological as well as weighted properties can be completely described by a weighted adjacency matrix **W**, whose elements *w*_*ij*_ denote the weight of the edge between node *i* and *j*. The definition of the BBV network is based on two coupled mechanisms: the topological growth and the weight dynamics, which is same as BA network.

(i) Growth. Starting from an initial small number of *N*_0_ nodes connected by edges with assigned weight *w*_0_, a new node is added at every time step. The new added node is connected to *m* different previously existing nodes with equal weight *w*_0_ for every edge and chooses preferentially nodes with large strength according to the probability ∏_*n*→*i*_ = *s*_*i*_/∑_*l*_ *s*_*l*_, where *s*_*i*_ is the node strength described as *s*_*i*_ = ∑_*j*_ *w*_*ij*_.

(ii) Weight dynamics. The weight of each new add edge is initially set to a given value *w*_0_ which is often set to 1 for simplicity. But the adding of edge connecting to node *i* will result in increasing the weight of the other edges linked to node *i* which is proportional to the edge weights. If the total increase is *δ* (we will focus on the simplest form: *δ*_*i*_ = *δ*), we can get

(1) $$\begin{array}{c} w_{ij}=w_{ij}+\Delta w_{ij}=w_{ij}+\delta *\frac{w_{ij}}{s_{i}}. \end{array}$$

This will yield the strength increase of node *i* as:

(2) $$\begin{array}{c} s_{i}=s_{i}+\delta +w_{0}. \end{array}$$

The degree distribution of BBV network *P*(*k*) ∝ *k* ^−*γ*_*k*_^ and the strength distribution *P*(*s*) ∝ *s* ^−*γ*_*s*_^ yield scale-free properties with the same exponent [6, 11, 21, 22]:

(3) $$\begin{array}{c} \gamma_{k}=\gamma_{s}=\frac{4\delta +3}{2\delta +1}=2+\frac{1}{2\delta +1}. \end{array}$$

Denoting *P*_*i*→*j*_ as the path between nodes *i* and *j* which pass through the nodes sequence *x*_0_( = *i*), *x*_1_, *x*_2_,…, *x*_*n*−1_, *x*_*n*_( = *j*), we define

(4) $$\begin{array}{c} F\left(P_{i\to j},\alpha \right)=\overset{n-1}{\underset{i=0}{\sum }}{w_{ij}}^{\alpha }. \end{array}$$

In our routing strategy, we specify the routing path between *i* and *j* as the one that makes *F*(*P*_*i*→*j*_, *α*) minimum under a given tunable parameter *α*.

In this paper, the simplest transfer model can be described as follows.

(1) All the nodes are treated as both hosts and routers. A host can create packets with addresses of destination and receive packets from other hosts while a router routes the data packets to their destinations.

(2) At each time step *t*, there are *R* packets generated in the whole network with randomly chosen sources and destinations. Once a packet is created, it is placed at the end of the queue if this node already has several packets waiting to be delivered to their destinations. The existing packets may be created at some previous time steps or they are transmitted from other nodes.

(3) At each time step, the first *C*_*i*_ packets at the top of the queue of each node *i*, if it has more than *C*_*i*_ packets in its queue, are forwarded one step toward their destinations and placed at the end of the queues of the selected nodes. Otherwise, all packets in the queue are forwarded one step. This procedure applies to every node at the same time.

(4) A packet, upon reaching its destination, is removed from the system.

In our model, three node delivery capability schemes are considered: (i) each node has the same packet delivery capability (*C*_*i*_ = const, CONC stands for this scheme); (ii) the node delivery capacity is considered to be proportional to the node degree *k*_*i*_ (*C*_*i*_ ~ *k*_*i*_, DEGC stands for this scheme); (iii) the node delivery capacity is considered to be proportional to the node strength *s*_*i*_ (*C*_*i*_ ~ *s*_*i*_, STRC stands for this scheme). To compare the overall transfer capacity, we assign the equal value to the total node delivery capability in three situations. In the last two schemes, we normalize the delivery capability of each node to set the total delivery capability of the whole network to be equal to the node number *n*, which is the same as the first case.

When *R* increases from zero to ∞, two phases will be observed: free flow for small *R* and congested phase for large *R*. We focus on the critical value *R*_*c*_ where phase transit from the former to the latter which can best reflect the maximum information transfer capacity of a system. For *R* < *R*_*c*_, the numbers of created and delivered packets are balanced, resulting in a steady free flow of traffic. For *R* > *R*_*c*_, traffic congestion occurs as the number of accumulated packets increases with time, due to the fact that the capacities of nodes for delivering packets are limited. We are interested in determining critical value *R*_*c*_ in order to address which kind of routing strategy is more susceptible to phase transition and therefore traffic congestion.

We introduce the betweenness *b*_*i*_ to estimate the possible packet passing through a node *i* under a given routing strategy which is defined as

(5) $$\begin{array}{c} b_{i}=\underset{s,t}{\sum }\frac{\sigma \left(s,i,t\right)}{\sigma \left(s,t\right)}, \end{array}$$

where *σ*(*s*, *i*, *t*) is the number of paths under the given routing strategy between nodes *s* and *t* that pass through node *i* and *σ*(*s*, *t*) is the total number of paths under the given routing strategy between nodes *s* and *t* and the sum is over all pairs *s*, *t* of all distinct nodes. The probability that a certain packet will pass through the node *i* is *b*_*i*_/∑_*j*=1_^*n*^ *b*_*j*_ (*n* is the total node number in the network). The average number of packets that the node *i* receives at each time step is *R*∗*b*_*i*_/(*n*∗(*n* − 1)). Congestion occurs when the number of incoming packets is equal to or larger than the outgoing packets that the node *i* can transfer at one time step; that is, *R*∗*b*_*i*_/(*n*∗(*n* − 1)) ≥ *C*_*i*_. So the critical packet generating rate *R*_*c*_ is

(6) $$\begin{array}{c} R_{c}=\min\left(C_{i}*n*\frac{n-1}{b_{i}}\right). \end{array}$$

In order to characterize the phase transition from free flow to congested phase, we use the order parameter introduced in [23]:

(7) $$\begin{array}{c} \eta =\underset{t\to \infty }{\lim}\frac{\langle \Delta \Theta \rangle }{R*\Delta t}, \end{array}$$

where ΔΘ = Θ(*t* + Δ*t*) − Θ(*t*), with 〈⋯〉 indicating average over time windows of width Δ*t*, and Θ(*t*) is the total number of packets in the network at time *t*. Therefore, in our simulation we can determine *R*_*c*_ as the phase transition point where *η* deviates from zero.

## 3. Simulation and Analysis

Since BBV networks have the same properties (i.e., the power-law distribution of degree, strength, and weight) as lots of real-world networks (the scientists collaboration networks, the Internet, and the WWW), we use BBV networks to investigate the network overall capacity.

In all simulations, we normalize the critical packet generating rate *R*_*c*_: we plot the normalized *R*_*c*_ and label *C*_*i*_ = 1 for the CONC scheme, *C*_*i*_ = *k*_*i*_/〈*k*〉 for the DEGC scheme, and *C*_*i*_ = *s*_*i*_/〈*s*〉 for the STRC scheme.

**Figure 1:**
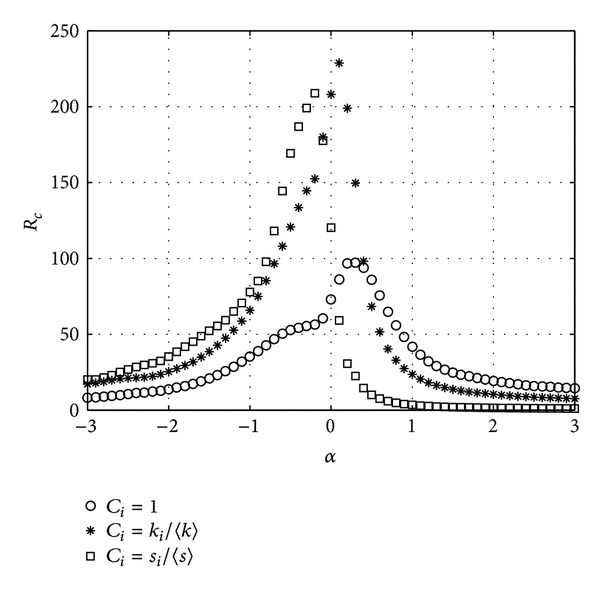
*R*_*c*_ versus *α*. BBV network with *n* = 1000, *δ* = 6, *m* = 6, and *ω*_0_ = 1.

In Figure 1, we plot the critical packet generating rate *R*_*c*_ versus different parameter *α* in a BBV network with *n* = 1000, *δ* = 6, *m* = 6, and *ω*_0_ = 1. (For every network, 20 instances are generated and for each instance, we run 20 simulations. The results are the average over all the simulations.)

From Figure 1, we can see that in the three schemes, the critical packet generating rate *R*_*c*_ varies with the tunable parameter *α*. In the CONC scheme, *R*_*c*_ reaches the peak when *α* is 0.3 while in the DEGC scheme and STRC scheme *α* is 0.1 and −0.2 correspondingly. The DEGC scheme where node delivery capability is proportional to its degree has the largest transfer capacity which is 2.35 times as the CONC scheme where node delivery capability is constant. And the transfer capacity of the STRC scheme is 2.15 times as the CONC scheme. Figure 1 show that when the node delivery capability is different, the BBV network has different transfer capacity which varies with the tunable parameter. The BBV network has the highest transfer capacity when the node delivery capability is proportional to its degree and the tunable parameter is 0.1.

**Figure 2:**
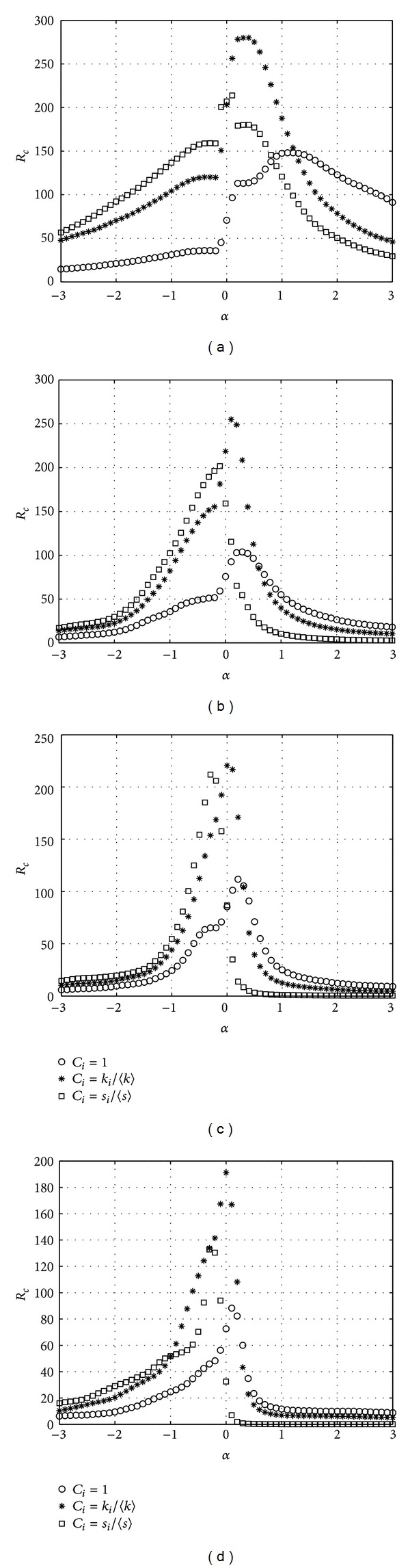
*R*_*c*_ versus *α*. BBV network with *n* = 1000, *m* = 6, and *ω*_0_ = 1. (a) *δ* = 0.6, (b) *δ* = 3, (c) *δ* = 12, and (d) *δ* = 60.

Then, we check the impact of *δ* on the *R*_*c*_. We set *δ* = 0.6, 3, 12, and 60 to get different simulation results in Figure 2.

As shown in Figure 2, all three schemes get their highest transfer capacity at different tunable parameter *α* because of different parameter *δ*. From formula (3), we can recognize that both the degree distribution and the strength distribution of BBV network are described by an exponent *γ* which depends on the parameter *δ*. When the parameter *δ* = 0, the BBV network is similar to BA network with *γ* = 3. And when the parameter *δ* increases, the distributions become broader with *γ* = 2 when the parameter *δ* → *∞* which results in different schemes obtaining peak transfer capacity at different tunable parameter *α*.

**Figure 3:**
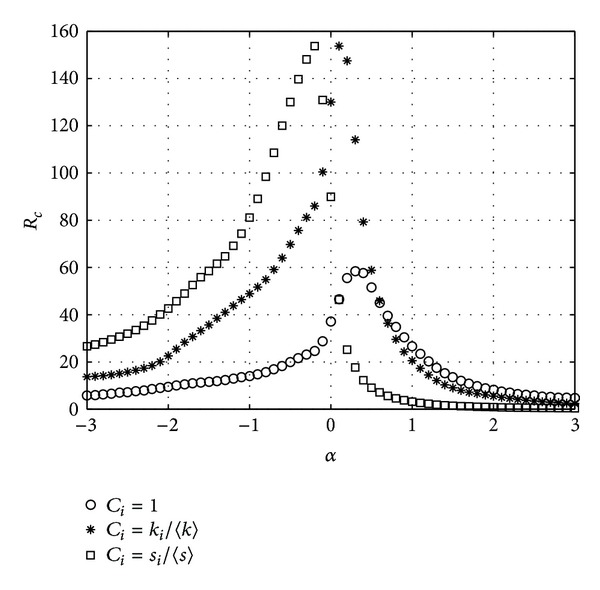
*R*_*c*_ versus *α*. BBV network with *n* = 1000, *δ* = 6, *m* = 3, and *ω*_0_ = 1.

**Figure 4:**
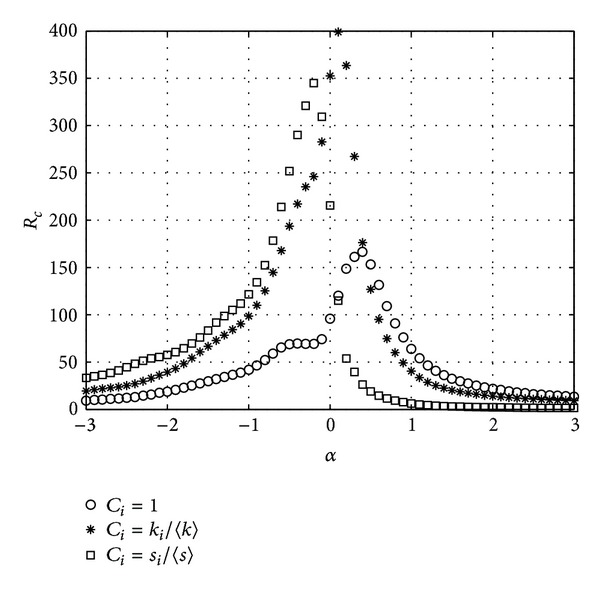
*R*_*c*_ versus *α*. BBV network with *n* = 2000, *δ* = 6, *m* = 6, and *ω*_0_ = 1.

Then we check the influence of the new add edges number *m* and the node number *n* on three different schemes. Simulation results are shown in Figures 3 and 4 correspondingly.

From Figures 3 and 4, we can come to the conclusion that the new add edges number *m* and the node number *n* have a little effect on the impact of tunable parameter *α* on the transfer capacity of three different schemes. They only affect the absolute value of the transfer capacity.

**Figure 5:**
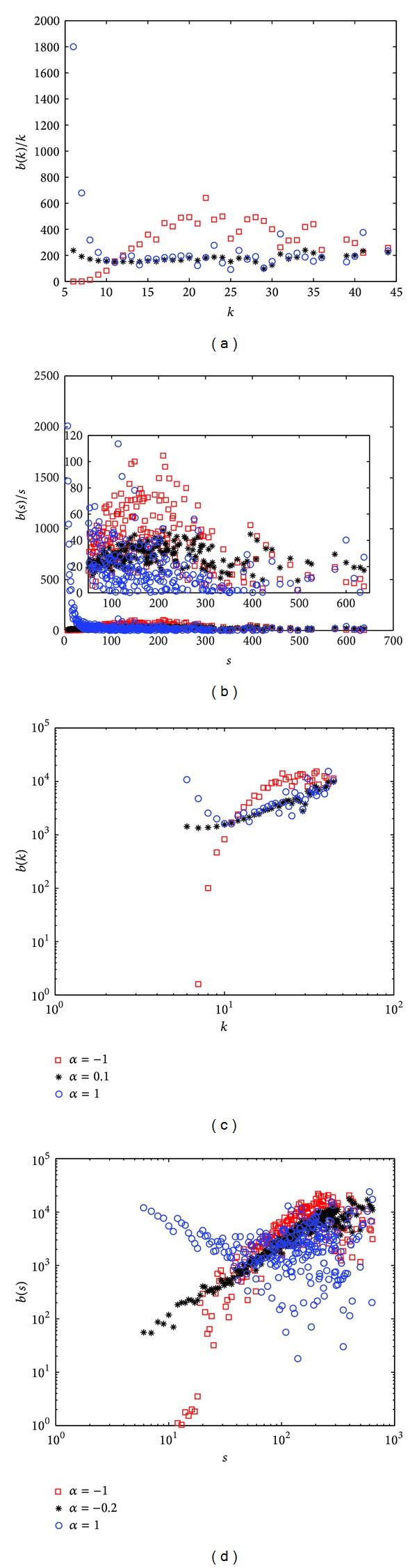
BBV network with *n* = 1000, *δ* = 6, *m* = 6, and *ω*_0_ = 1. (a) DEGC, betweenness per node. (b) STRC, betweenness per node. (c) DEGC, betweenness distribution versus node degree. (d) STRC, betweenness distribution versus node strength.

By investigating the betweenness distribution on the network, a heuristic explanation for the optimal tunable parameter *α* corresponding to the highest transfer capacity is presented in Figure 5.

The results of DEGC scheme are shown in Figures 5(a) and 5(c). In both figures, when the tunable parameter *α* is 0.1, the load is distributed more evenly than the other two. In Figure 5(a), the betweenness divided by node degree is relatively flat which means that the node with higher degree forward more packets. And in Figure 5(c), the linear characteristic in the log-log plot expresses the same meaning. The results of STRC scheme shown in Figures 5(b) and 5(d) are of the same meaning while the tunable parameter *α* is −0.2.

**Figure 6:**
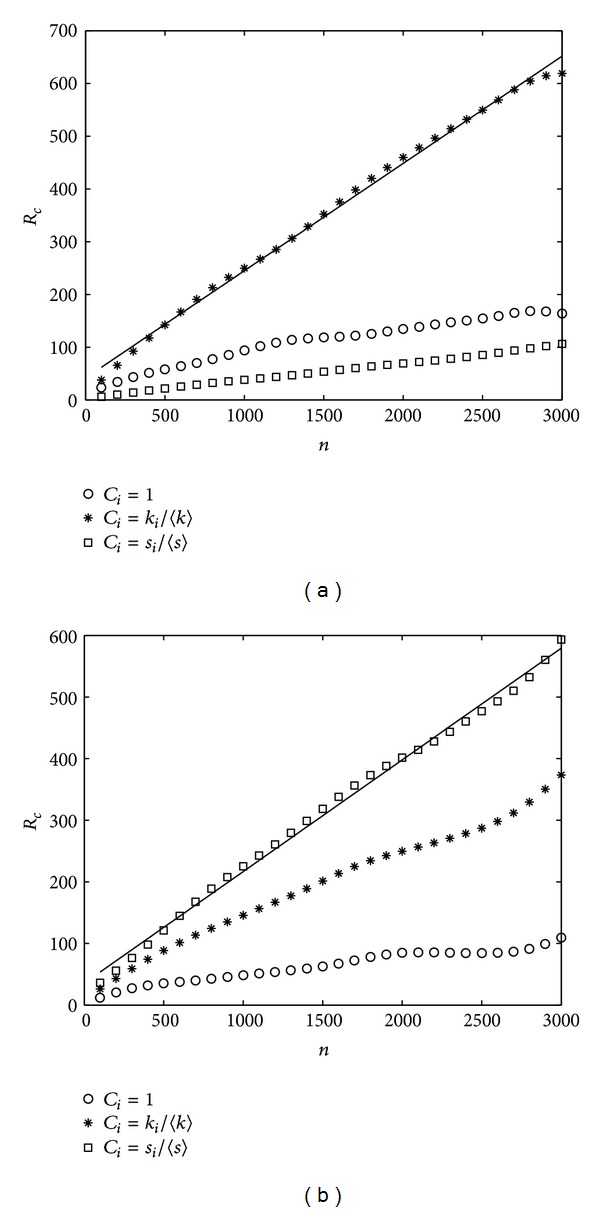
*R*_*c*_ versus *n*. BBV network with *δ* = 6, *m* = 6, and *ω*_0_ = 1. (a) *α* = 0.1, (b) *α* = −0.2.

In Figure 6, we show the relationship between the critical packet generating rate *R*_*c*_ and the node number *n*.  Figure 6(a) indicates that the network capacity of the DEGC scheme is always much larger than those of the STRC and CONC schemes when the tunable parameter *α* is 0.1. And Figure 6(b) indicates the situation of the STRC scheme with *α* = −0.2.

**Figure 7:**
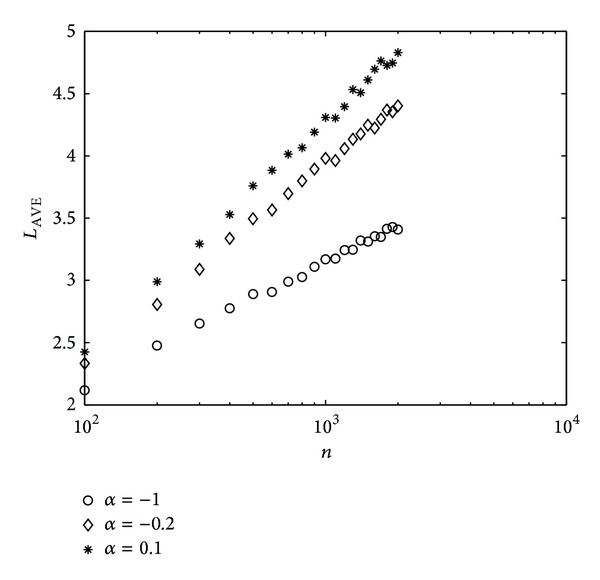
*L*_AVE_ versus *n*. BBV network with *δ* = 6, *m* = 6, and *ω*_0_ = 1.

The average weighted average length [24] *L*_AVE_ versus the node number *n* is reported in Figure 7. Although the weighted average length of DEGC scheme with *α* = 0.1 and the STRC scheme with *α* = −0.2 are higher than that of the traditional shortest path with *α* = −1, the small-world phenomenon, that is, *L*_AVE_ ∝ ln⁡*n*, is still maintained.

**Figure 8:**
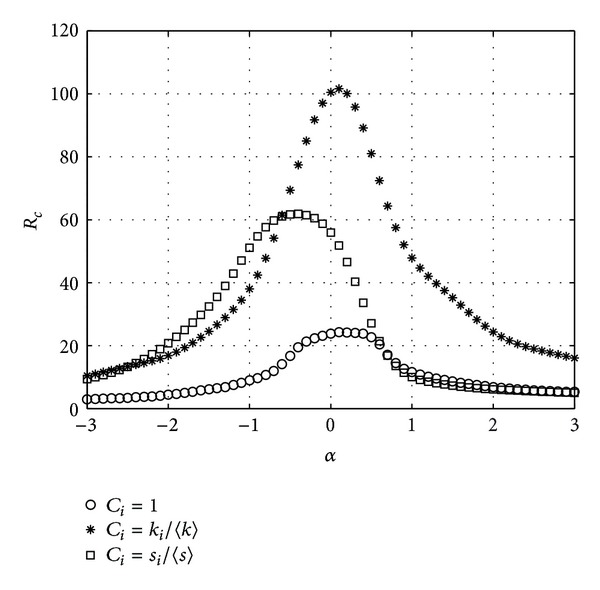
*R*_*c*_ versus *α*. Real-world network.

Finally, we test the three schemes on real-world networks. We choose the scientific collaboration network [25] which has a giant component of 5835 nodes. Simulation results are shown in Figure 8.

From Figure 8, we can see that in CONC scheme the network has the maximum transfer capacity, 24.2, when the tunable parameter *α* is near 0.3. In DEGC scheme the peak value is 101.6 with *α* = 0.1 and in STRC scheme it is 60.5 with *α* = −0.2. It means our strategy also works well in the real world network.

## 4. Conclusion

Considering the different node transfer capability, this paper has proposed a new routing strategy to enhance the network transfer capacity in weighted networks. The characteristic of our strategy is to select the optimal routing path according to three kinds of different schemes. The simulation yields some results different from those of previous studies. In most cases, the optimal value of the tunable parameter is 0.3 in the scheme in which each node has the same packet delivery capability (CONC), 0.1 in the scheme in which the node delivery capacity is considered to be proportional to the node degree (DEGC), and −0.2 in the scheme in which the node delivery capacity is considered to be proportional to the node strength (STRC). It is worth mentioning that in some weighted network the optimal value fluctuate around the mentioned value. And the scheme in which the node delivery capacity is proportional to the node degree has the highest transfer capacity when the tunable parameter is −0.2. At last, we apply our routing strategy on the scientific collaboration network to show the validity of the strategy on real-world networks. Moreover, the above-mentioned research may be practically useful for designing communication protocols.
